# Practical potential of suspension electrodes for enhanced limiting currents in electrochemical CO_2_ reduction†

**DOI:** 10.1039/d3ya00611e

**Published:** 2024-03-15

**Authors:** Nathalie E. G. Ligthart, Gerard Prats Vergel, Johan T. Padding, David A. Vermaas

**Affiliations:** a Department of Chemical Engineering, Delft University of Technology 2629 HZ Delft The Netherlands D.A.Vermaas@tudelft.nl; b Department of Process and Energy, Delft University of Technology Leeghwaterstraat 39 2628 CB Delft The Netherlands

## Abstract

CO_2_ conversion is an important part of the transition towards clean fuels and chemicals. However, low solubility of CO_2_ in water and its slow diffusion cause mass transfer limitations in aqueous electrochemical CO_2_ reduction. This significantly limits the partial current densities towards any desired CO_2_-reduction product. We propose using flowable suspension electrodes to spread the current over a larger volume and alleviate mass transfer limitations, which could allow high partial current densities for CO_2_ conversion even in aqueous environments. To identify the requirements for a well-performing suspension electrode, we use a transmission line model to simulate the local electric and ionic current distributions throughout a channel and show that the electrocatalysis is best distributed over the catholyte volume when the electric, ionic and charge transfer resistances are balanced. In addition, we used electrochemical impedance spectroscopy to measure the different resistance contributions and correlated the results with rheology measurements to show that particle size and shape impact the ever-present trade-off between conductivity and flowability. We combine the modelling and experimental results to evaluate which carbon type is most suitable for use in a suspension electrode for CO_2_ reduction, and predict a good reaction distribution throughout activated carbon and carbon black suspensions. Finally, we tested several suspension electrodes in a CO_2_ electrolyzer. Even though mass transport limitations should be reduced, the CO partial current densities are capped at 2.8 mA cm^−2^, which may be due to engineering limitations. We conclude that using suspension electrodes is challenging for sensitive reactions like CO_2_ reduction, and may be more suitable for use in other electrochemical conversion reactions suffering from mass transfer limitations that are less affected by competing reactions and contaminations.

## Abstract 2

Poor mass transfer in aqueous electrochemical CO__2__ reduction limits the achievable current density toward desired products. We introduce flowable suspension electrodes to spread the current over a larger volume and alleviate mass transfer limitations.

## Graphical abstract

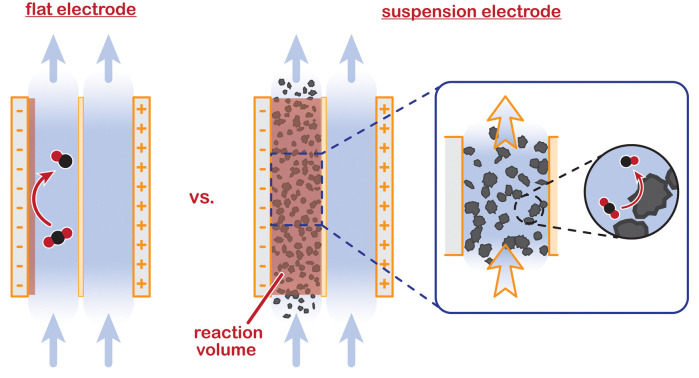

## Introduction

The high level of carbon dioxide (CO_2_) in our atmosphere is causing notable climate change all over the world, and levels are still rising. We need to significantly lower fossil fuel emissions by transitioning towards clean energy, in order to mitigate climate change.^1^ The most familiar and popular choice of renewable energy is green electricity, but this cannot power all processes. For some applications this is due to intermittency of wind and sunlight, while other sectors cannot run on electricity and are likely to remain dependent on hydrocarbons (*e.g.* cargo ships, planes, plastics and pharmaceuticals).^2^

We can introduce CO_2_ circularity by using renewable, synthetically produced hydrocarbons to replace fossil fuels.^2–4^ Modern technologies can extract CO_2_ from the air^5^ or ocean,^6^ after which the CO_2_ can be converted into fuels or chemicals. Electrochemical CO_2_ reduction is widely studied as a conversion method because it requires only CO_2_, water and electricity as input. Nevertheless, CO_2_ electrolysis is only commercially viable when operating at high current densities of at least 200 mA cm^−2^.^2,7^

The current density at which CO_2_ can be converted is limited by the availability of CO_2_ at the catalyst surface.^8–10^ Because CO_2_ has a low solubility in water (34 mM, at ambient temperature and pressure^11^), even low current densities cause CO_2_ depletion at the electrode surface in aqueous reactors,^11,12^ while the remaining current drives the hydrogen evolution reaction (HER).^3,8,9,13^ This limits the maximum CO partial current density to about 2 mA cm^−2^ in aqueous systems that rely on forced convection and diffusion.^14^ Bubble-induced mixing^15^ and leveraging buffering reactions with bicarbonate^16,17^ can raise this up to tens of mA cm^−2^, which is still well below the required 200 mA cm^−2^. Therefore, our challenge is to accelerate CO_2_ mass transport towards the electrode.

Several strategies to enhance mass transport have been investigated, each with their own advantages and challenges. The most widely applied strategy is to supply CO_2_ in gas phase instead of dissolved in an electrolyte. Examples of such electrolyzers are flow cells with a gas diffusion electrode (GDE),^18,19^ membrane electrode assemblies (MEAs)^20–22^ and solid oxide electrolysis cells (SOECs).^23–25^ Using a gaseous CO_2_ supply significantly raises the CO_2_ flux towards the electrode surface and boosts the limiting current density. Although this concept is promising, vapour-fed electrolyzers are delicate and complicated systems. Challenges in scaling up include water management at the porous electrode^26,27^ and drying out of ionic separators.^28,29^ Additionally, stability issues occur due to differential pressure and electrowetting,^26,27,30^ salt formation^8^ and degradation of carbon in the porous electrode.^30^ These complications in GDE-based CO_2_ electrolyzers raise the question whether there are still unexplored strategies to circumvent the mass transfer limitations in aqueous CO_2_ reduction.

We propose to use suspension electrodes to alleviate mass transfer limitations in CO_2_ electrolyzers and boost the achievable CO_2_ reduction current density. In suspension electrodes, electric charges are transported into the bulk of the electrolyte by conductive networks of microparticles, or their capacitive functionality.^31^ Using a suspension electrode brings several potential advantages over using a conventional configuration, including (1) the use of dissolved CO_2_ in the full volume instead of a thin layer at the cathode, (2) a lower local current density inside the suspension because of the large surface area, and (3) flowing microparticles may induce additional mixing of the electrolyte. While suspension electrodes have been studied for various applications, including flow capacitors,^32,33^ flow batteries,^34–36^ deionization technology^37,38^ and microbial fuel cells,^39,40^ they have not been applied in electrochemical CO_2_ reduction. The conductivity and capacitance can be tuned through material choice, particle loading, or addition of conductive additives. High surface area carbon materials have high capacitance, but are usually less conductive than graphitic carbons with lower surface area.^41^ The effects of suspension material and loading, and the associated conductive networks, capacitance and viscosity are yet unknown in CO_2_ electrolyzers.

In this work, we identify the requirements for a well-performing suspension electrode for electrochemical CO_2_ reduction. We do this by measuring important suspension properties, including electric conductivity and viscosity. We use the results to model local current densities inside the electrolyzer channel and find the key parameters that determine when a suspension is used to its full advantage. Finally, we test several suspension electrodes in a CO_2_ electrolyzer. Our findings can help in adapting the composition of suspension electrodes for use in mass transfer limited electrochemical processes.

## Concept

**Fig. 1:**
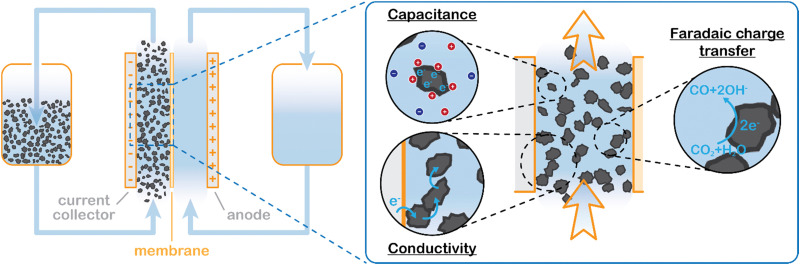
Suspension based electrochemical flow system (left) and charge transfer mechanisms inside the flowing suspension electrode (right). Charge transfer takes place through electric double layer charging (capacitive charge storage), electric conductivity *via* particle networks, and faradaic charge transfer, in this example CO_2_ reduction to CO.

We propose to combine a CO_2_ reduction flow cell with an electrocatalytic suspension electrode. In such a configuration, the flow cell consists of two flow channels through which electrolyte is pumped continuously. The compartments are separated by an ion exchange membrane. Our concept makes use of a relatively inert current collector (such as glassy carbon or graphite), while the CO_2_ reduction reaction takes place at the surface of suspended microparticles. A schematic representation of such a system is shown in Fig. 1.

The suspension electrode should consist of electrically conductive and capacitive microparticles (typically between 5 and 20 wt%^42^) that are suspended in an electrolyte. A current is applied to the suspension *via* a current collector and conducted into the bulk *via* particle networks.^31^ Electric double layer (EDL) formation facilitates charge storage inside a particle when it temporarily detaches from a network.^31^ This capacitive effect enables the particle to transfer the charge further into the suspension or continue the reaction. A schematic of the charge transfer mechanisms is shown in Fig. 1.

Suspension electrodes can be designed for many applications because their properties and functionality rely on their composition.^41^ For example, highly porous carbon particles are well-suited for use in applications that rely on high capacitance, such as electrochemical flow capacitors (EFCs) and flow electrode capacitive deionization (FCDI), while redox active materials can be added to make a redox flow battery (RFB).^35,37^ Suspension electrodes have been shown to work well in microbial fuel cells (MFCs) as well. MFCs benefit significantly from the large surface area provided by the microparticles.^39,40^ The increased surface area allows for lower local current densities and higher capacitance. The EDL acts as electron supply for the microbes while they are not in contact with the current collector, and thus allows for longer reaction time. We expect to see the same advantage in mass transfer limited reactions, like aqueous CO_2_ reduction.

Having high electric and ionic conductivity, and low viscosity are important for minimizing Ohmic and pumping losses.^41^ Although raising the particle loading significantly enhances both electrical conductivity and capacitance, it also considerably increases viscosity and thus decreases the flowability of the system.^43,44^ Alternatively, conductive additives can be added in low amounts (up to 5 wt%) to boost conductivity. Depending on material, size and shape, some microparticles and additives have a lower impact on viscosity.^45^ However, achieving both good electrical and good rheological properties in one suspension remains challenging.

As mentioned in the introduction, we expect higher limiting current densities in suspension electrodes because of three principles. First, using a suspension electrode allows for the current to percolate through the whole flow channel, making CO_2_ in the whole channel volume available for reduction. We can estimate how much additional CO_2_ is made available for reaction in our suspension cell compared to a plate electrode. For our channel thickness of 3 mm, and CO_2_ concentration of 34 mM, the compartment contains 10 μmol of CO_2_ per (geometric) cm^2^. Assuming that the electric current in a suspension can reach the full compartment thickness and that he inter-particle distance is smaller than the boundary layer thickness (typically 100 μm), the complete 10 μmol of CO_2_ per cm^2^ is available for reaction. In contrast, a plate electrode has charge transfer only at the boundary of the channel and CO_2_ molecules need to travel towards it before they can be converted. In this case, we need to consider the slow transport across the diffusion boundary layer. We estimate the amount of CO_2_ transported to the electrode per second (*ṅ*) from 
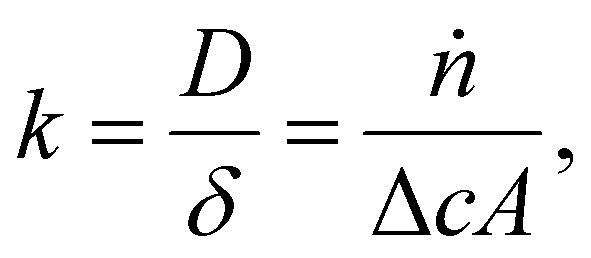
 with a mass transfer coefficient (*k*) in the order of 10^−5^ m s^−1^ for a diffusion coefficient (*D*) of 10^−9^ m^2^ s^−1^ and a diffusion layer thickness (*δ*) of 100 μm,^46,47^ and a concentration difference between the surface and bulk (*c*) of 34 mM, on an area (*A*) of 1 cm^2^. This results in only 0.2 μmol cm^−2^ being able to reach the flat electrode during a residence time of 5 s. This is 50 times less than the 10 μmol of CO_2_ that can be reached by the suspension electrode. Hence, suspension electrodes could increase the limiting current density by a factor 50. In addition to having more CO_2_ available due to the larger reaction volume, the applied current density is spread over a significantly larger surface area and the local current density can be lowered by an order of magnitude compared to the geometrical current density. This lowers the required charge transfer overpotential and promotes selectivity towards the desired reaction.^48^ Finally, solid phase particles have been shown to induce mixing in the liquid phase in two-phase flows.^49^ This can further accelerate CO_2_ mass transfer towards the catalytic surface.

## Methods

### Modelling

**Fig. 2:**
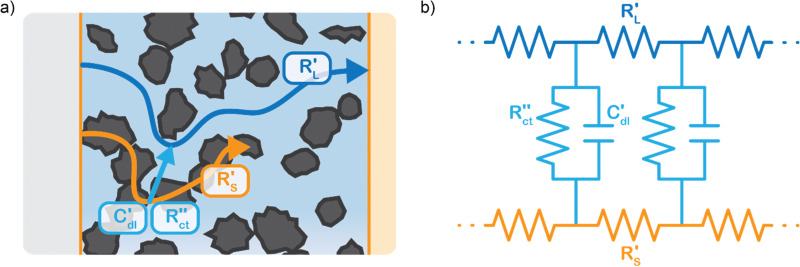
(a) Schematics of the charge transfer pathways through the liquid, solid and interface of a suspension electrode, and (b) the corresponding equivalent circuit used in the TLM model. The ionic and electric conductances are described using their resistances 
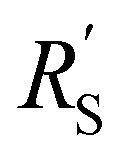
 and 
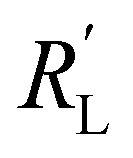
, respectively, while the interfacial charge transfer consists of a capacitive EDL 
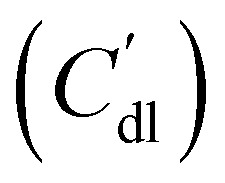
 and faradaic charge transfer 
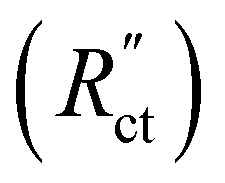
.

We model the solid and liquid phase currents throughout the channel to evaluate where the reaction is taking place in suspensions of different particle types and loadings, and electrolyte concentrations. We consider the suspension as a porous electrode and use the transmission line model (TLM) by Alfisi *et al.* with the corresponding equivalent circuit shown in Fig. 2b.^50^ The model considers two charge transfer pathways, through the solid and liquid phases with resistances (per unit length, Ω cm^−1^) 
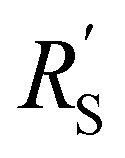
 and 
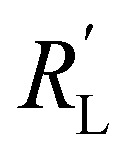
, respectively as shown in Fig. 2a. We use the solid resistance extracted from electrochemical impedance spectroscopy (EIS) measurements in the next section (Experiments) to account for the temporality and changeability of the porous network in the suspension. The interfacial impedance between solid and liquid phase consists of a volumetric charge transfer resistance 
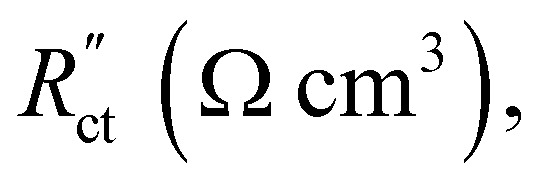
 which results in the faradaic current, and a volumetric double layer capacitance 
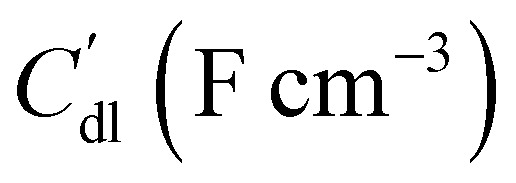
 in parallel.^50^

The following governing eqn (1) and (2) are found by defining the potential drops over infinitesimal elements in the liquid and solid phase, respectively, and linking them through the interfacial impedance:^50^1
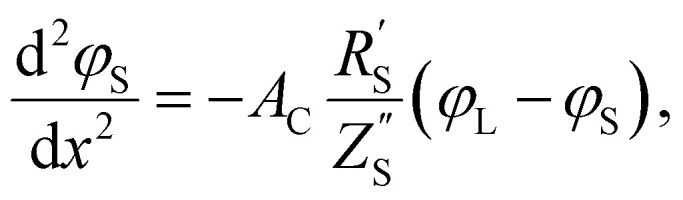
2
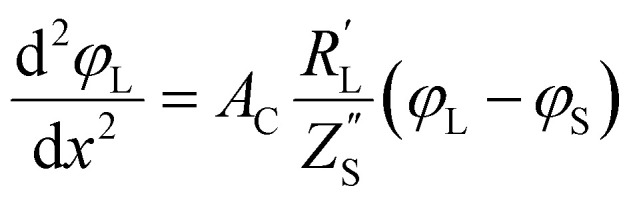
in which3
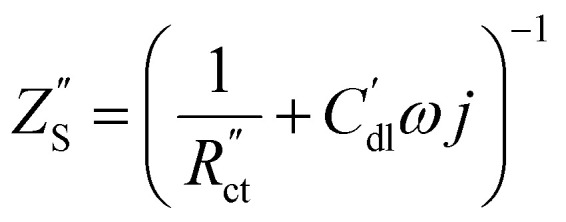


Here *A*_C_ is the cross-sectional area of the channel (cm^2^) and the *x*-direction is taken to be across the flow channel, ranging from *x* = 0 at the current collector to *x* = *l*_e_ at the membrane. We set the potential at *x* = 0 to be the applied potential (*V*_app_), and assume a completely ionic current at the membrane, resulting in boundary conditions^50^4
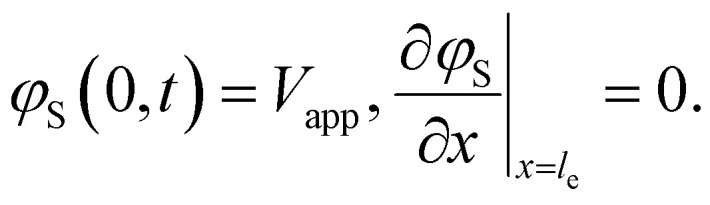


Additionally, we set the liquid potential at the membrane to 0, and assume a completely electric current at the electrode interface, yielding^50^5
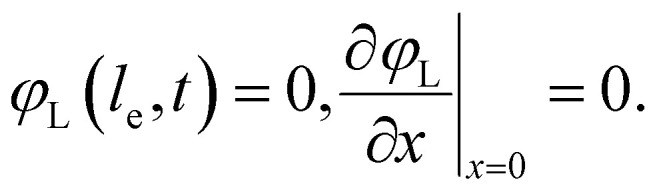
With these boundary conditions, we solved the governing eqn (1) and (2) numerically for low frequency *ω* to approximate DC voltages.

### Experiments

The slurries were prepared by adding carbon material to 0.5 M KHCO_3_ (≥99%, ThermoFisher Scientific) as a typical electrolyte for CO_2_ reduction,^9,51^ under stirring. The suspensions were sonicated (ultrasonic cleaner USC 500 TH, 45 kHz, VWR) for 30 minutes. The slurries consisted of 0–20 wt% activated carbon (AC, 20 μm median particle size, 1000 m^2^ g^−1^, Norit SX Plus CAT, Sigma Aldrich), carbon black (CB, average particle size of 50 nm, 250 m^2^ g^−1^, Vulcan XC-72, fuel cell store), or 0–40 wt% glassy carbon spheres (gC, 10–20 μm glassy carbon spherical powder, Alfa Aesar). In the suspensions used for electrolysis, 25 wt% of the solid content was replaced by Ag nanopowder (20–40 nm, 99.9%, Alfa Aesar) to function as catalyst.

Rheology measurements were performed on carbon suspensions without Ag nanopowder using a stress controlled dynamic hybrid rheometer (TA Instruments, DHR-3). The rheometer was equipped with a Couette geometry consisting of a stainless steel cup (diameter of 30 mm) with Peltier heating element and stainless teel DIN rotor (28 mm diameter, 42.07 mm length). All measurements were performed while maintaining a gap of 5917.1 μm between the rotor and the bottom of the cup, and a temperature of 25 °C. The shear rates of interest ranged between 2 and 1000 s^−1^ and were applied for 3–4 minutes. The suspension was pre-sheared at 2000 s^−1^ before each measurement to erase memory and sedimentation effects.^52^

The suspension impedance was measured under flow conditions in a custom-made flow cell (Fig. S9, ESI†), incorporating only one flow channel (3 mm thick PMMA) and no ion-exchange membrane. The slurries were pumped (peristaltic L/S precision bump system, Masterflex) upwards through the channel between two graphite (99.95% rigid graphite, Goodfellow) current collectors with four electrical connections. EIS was performed with an Autolab potentiostat (PGSTAT302N, Metrohm). A sinusoidal perturbation with a frequency range from 0.1 to 10^5^ Hz was applied with an amplitude of 5 mV around the open circuit voltage (OCV).

**Fig. 3:**
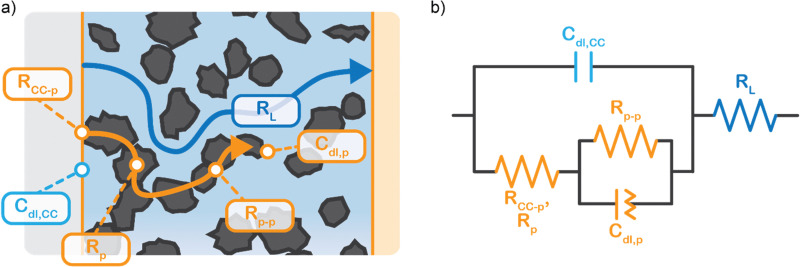
Schematics of the (a) non-faradaic charge transfer pathways in a suspension electrode taking place near the open circuit voltage (OCV) and (b) the equivalent circuit containing the corresponding electrical elements that was used for fitting the EIS data. The current is transferred between the current collector to particles *via* a resistance *R*_CC–p_. The particles have a material resistance *R*_p_, an interparticle resistance *R*_p–p_, and an imperfect capacitance *C*_dl,p_ that can be fitted with a constant phase element (CPE). The current collector capacitance and electrolyte resistance are fitted as *C*_dl,CC_ and *R*_L_, respectively.

EIS provides insight into properties, such as conductivity and capacitance, of different processes in electrochemical systems. These can be extracted by fitting the EIS data to an equivalent circuit of the system. Because we run the EIS in a potential window with only non-faradaic reactions, the equivalent circuit deviates from that in Fig. 2b. A schematic of important processes in suspension electrodes is shown in Fig. 3a, and can be used to deduce a sensible equivalent circuit. The current applied to the current collector can take various paths, namely it can charge the EDL, with a capacitance *C*_dl,CC_, and proceed as ionic current through the electrolyte with a resistance *R*_L_. Alternatively, the current can be electrically conducted into the suspension *via* a contact resistance between the current collector and a particle (*R*_CC–p_), after which the current travels through the suspension *via* particle networks and collisions. These consist of the carbon material resistance (*R*_p_) and contact resistance between particles (*R*_p–p_). Instead of transferring to another particle, electrons can be stored in the EDL at a particle surface, which can be described as an imperfect capacitance (*C*_dl,p_) in a constant phase element (CPE). We combined the electrical elements corresponding to these processes into the equivalent circuit shown in Fig. 3b and used this to fit the EIS data.

Electrolysis was performed *via* chronopotentiometry in the same suspension flow cell equipped with two flow channels (as shown in Fig. S9, ESI†) separated by a Selemion anion exchange membrane (100 μm, AGC engineering) that was pre-soaked in electrolyte. A graphite current collector, an Ir-/Ru-oxide coated Ti-sheet (Permascand) anode, and a leak-free Ag/AgCl reference electrode (LF-1-45, Alvatek) were used for electrolysis. Both the catholyte (suspension) and anolyte (0.5 M KHCO_3_) were saturated by sparging 50 mL min^−1^ CO_2_ for at least 30 minutes before, and continuously purged and recirculated (peristaltic L/S precision pump system, Masterflex) during each experiment. A constant current density was applied with a an IviumStat.h (±5 A/±10 V, Ivium) for 45 minutes, during which samples of the product gases were taken every 3–4 minutes from the headspace of the catholyte reservoir and analyzed with an inline gas chromatograph (CompactGC4.0, Interscience).

## Results and discussion

### Ratio of reaction and conduction resistances is key in electrode utilization

We modelled the local current densities for different ratios of 
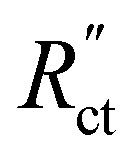
 with 
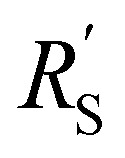
 and 
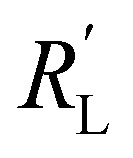
 to evaluate the influence on electrode utilization and reaction distribution. The current densities in the solid and liquid phases are calculated with eqn (6) and (7) respectively.^50,53^6
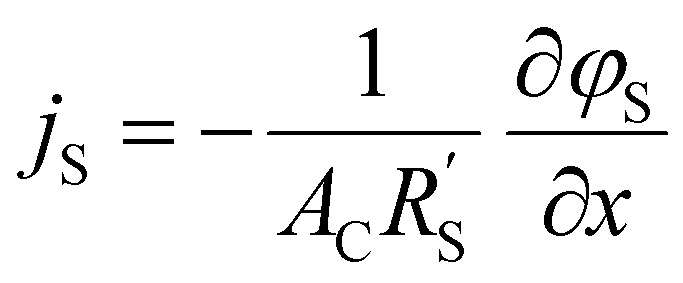
7
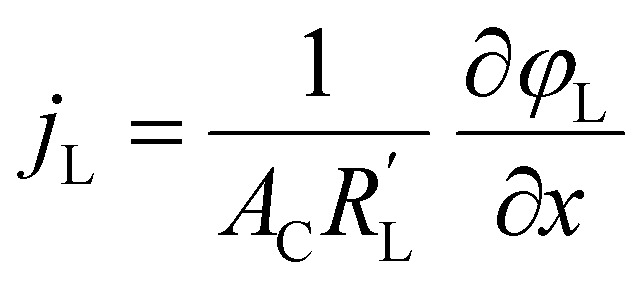


**Fig. 4:**
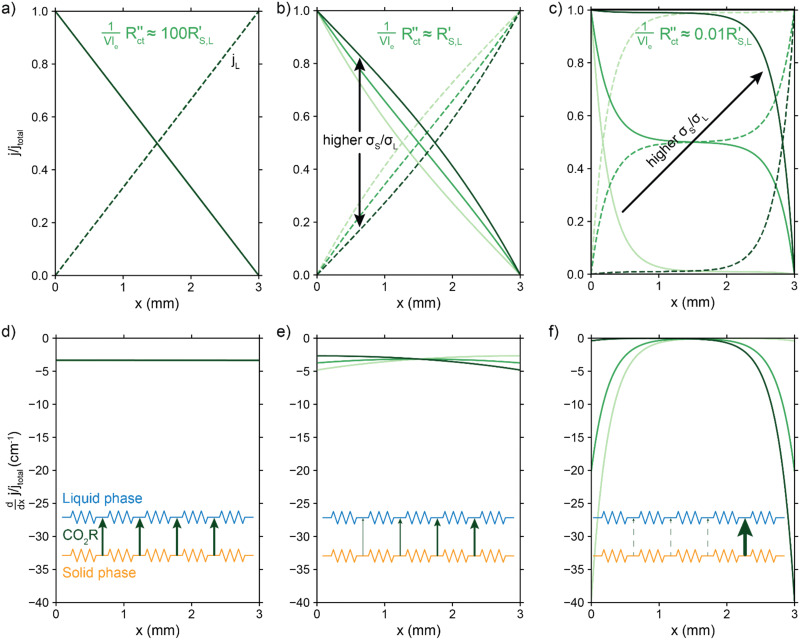
Modelled local currents throughout the electrolyzer channel for different ratios of solid and liquid conductivities and charge transfer resistance. Normalized solid and liquid currents (top figures), and the slope (bottom figures) of the solid current fraction that indicates the interfacial current. A schematic representation of the TLM circuit is displayed at the bottom with the arrows indicating the intensity of the interfacial current and reaction in that region. We show the results for different ratios of solid (*σ*_*S*_) and liquid (*σ*_*L*_) phase conductivities. (a) and (d) 
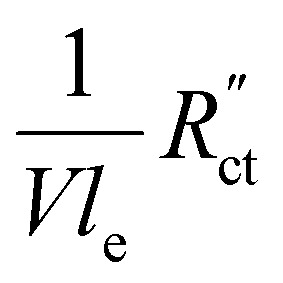
 is a factor 100 higher than, (b) and (e) the same magnitude as, (c) and (f) and a factor 100 lower than 
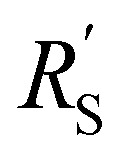
 and 
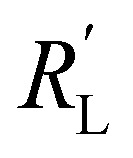
.

Fig. 4a–c show the relative contributions to the current that are conducted through the solid (*j*_S_/*j*_total_) and liquid (*j*_L_/*j*_total_) phase, at different ratios of 
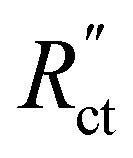
 with 
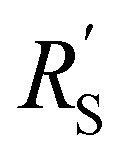
 and 
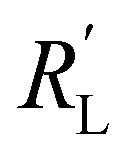
. A factor of 
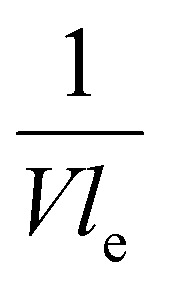
is included to match the units and allow for comparison of the values, where *V* and *l*_e_ are the electrode volume and thickness, respectively. The derivation of this factor is included in the ESI.† This factor depends on the geometry of the cell, and is close to unity for our case (*V* = 2.5 cm^3^, *l*_e_ = 0.3 cm). Fig. 4d–f indicate the local charge transfer from the solid to the liquid phase over the thickness of the channel.

When the normalized charge transfer resistance 
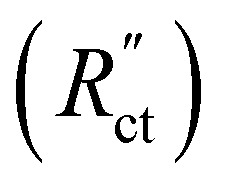
 is much higher than the resistance of the solid and liquid phases (Fig. 4a and d), the reaction distributes evenly over the full channel. Consequently, the current through the solid phase decreases linearly with increasing distance from the current collector, while the current through the liquid accumulates linearly (Fig. 4a). Hence, the faradaic current is constant throughout the channel (Fig. 4d). Such a case resembles a suspension electrode with the reaction occurring over the full channel thickness.

The situation changes slightly when 
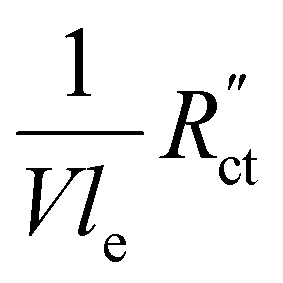
 is in the same order of magnitude as the solid 
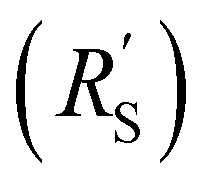
 and liquid 
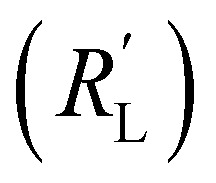
 phase resistances, as shown in Fig. 4b and e. In this case, the faradaic current can still be relatively equally distributed, but the ratio between 
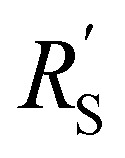
 and 
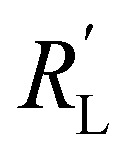
 gains importance and determines at which side of the channel the reaction is favoured. The system minimizes the total resistance, causing the current to be carried longer in the phase with the lowest resistance. For example, when the solid resistance is low, the current tends to transfer from the solid to the liquid phase later in the channel, pushing the main reaction location towards the membrane (*x* = 3 mm). Oppositely, the faradaic charge transfer occurs dominantly near the current collector (*x* = 0 mm) in case of a higher solid phase resistance.

This effect is especially visible when 
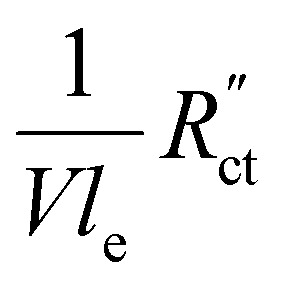
 is significantly lower than either phase resistance (Fig. 4c and f), in which case the reaction only occurs at the sides of the channel. For small 
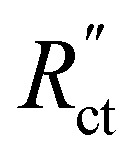
, the interfacial current is divided over the current collector and membrane region only when 
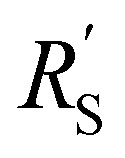
 and 
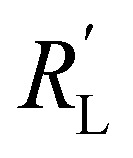
 are equal, but is otherwise localized at one side. Either situation gives a relatively high local interfacial current, which does not optimally leverage the suspension electrode concept and thus will not help to alleviate mass transfer limitations.

This means that the ratio between 
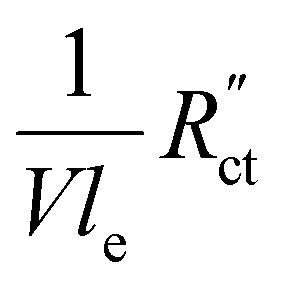
 and 
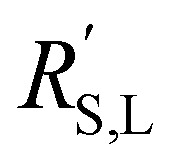
 is crucial for spreading the reaction over the whole channel and utilizing the suspension electrode to its full advantage. The suspension electrode would work well in case of a sluggish reaction, or in case of highly conductive solid and liquid phases that ensure that the faradaic charge transfer is the dominant resistance. Alternatively, the solid and liquid phase resistances should be well-matched whenever they near the charge transfer resistance.

### Particle size and shape impact conductivity and flowability trade-off

As seen from the model, achieving electric and ionic conductivity that are sufficiently high to compete with the faradaic charge transfer is essential for optimizing suspension electrodes. However, producing a high electric conductivity of the suspension with good flowability is a well-known challenge.^43,44^ Raising the carbon loading is the most effective method for improving conductivity, but it also significantly lowers the flowability. However, we hypothesize that even though both conductivity and viscosity have a relation to enhanced particle–particle interaction, the relation is not necessarily linear and may differ for different materials.^54^ Because both properties are highly particle-dependent, we measured the viscosity and conductivity of the three particle types used in this study. We combine the data to determine which particle type has the most favourable flowability-conductivity relation.

**Fig. 5:**
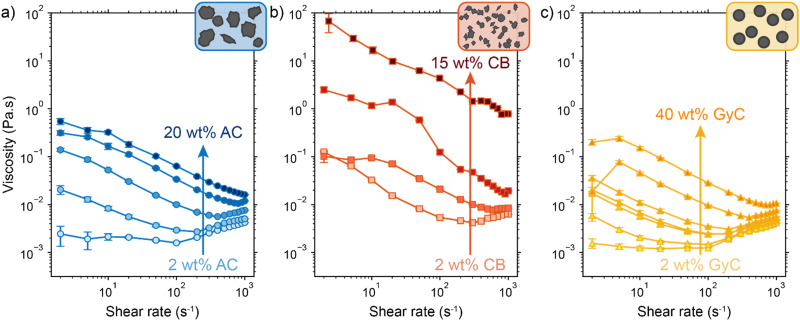
Measured viscosity for different shear rates and carbon loadings of (a) activated carbon (AC), (b) carbon black (CB), and (c) glassy carbon spherical (GyC) suspensions. The inserts show an impression of the differences in size and shape between the particle types. We see significantly higher viscosities in smaller and irregularly shaped carbons.

The rheology results for activated carbon (AC, 2–20 wt%), carbon black (CB, 2–15 wt%) and glassy carbon spheres (GyC, 2–40 wt%) suspensions are shown in Fig. 5. Whereas the slurries of all particle types show shear thinning behaviour, we see a large difference in viscosity of several orders of magnitude. The irregularly shaped particles (AC and CB) cause significantly higher viscosity than the spherical particles (GyC) at the same loading. The CB suspension, which contains the smaller of the two irregularly shaped particle types, is the least flowable; this material displays such a high viscosity and paste-like consistency at 20 wt% that the sample could not be tested. The glassy carbon spheres show a considerably lower viscosity, with the most viscous GyC suspension of 40 wt% approximately matching the 15 wt% AC slurry. In addition to being of approximately the same size as the AC particles, the glassy carbon particles have a spherical shape with a smooth surface. This makes the contact areas between the particles smaller and the smoothness of the surface imposes less friction during a collision.^55^ Our observations that smaller size and a more irregular shape cause higher viscosity in the carbon suspensions is in good agreement with existing literature.^55^

**Fig. 6:**
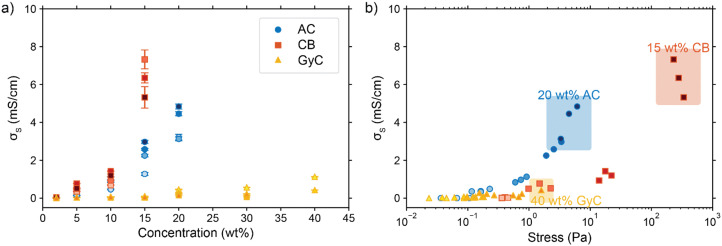
Measured conductivities and their dependence on (a) carbon loading for AC, CB, and GyC suspensions (darker colours indicate higher flow rates, error bars give the error in the EIS fit) and on (b) stress as a result of varying viscosity and shear rates (shear rates between 15 and 75 s^−1^, as relevant for electrolysis experiments). The results show an increase in conductivity with loading for all carbon types. The relation between conductivity and stress is highly dependent on particle type and most favourable for AC suspensions.

Next, we consider the experimentally obtained electric conductivities in Fig. 6a for all different concentrations of the various particles. As could be expected from the viscosity results, the glassy carbon spheres show the lowest conductivity due to lack of inter-particle contact. Following the same train of thought and considering the large difference that was observed in viscosity between AC and CB, it is surprising that both suspension types show similar conductivity up to a concentration of 10 wt%, while CB surpasses AC only at a loading of 15 wt%. The sharp increase in conductivity between 10 and 15 wt% of CB suggests that the critical concentration for forming extensive percolation networks lies in this region.^31,56^ From this graph, one could select CB as the most conductive particle type. However, we should keep in mind that this carbon type also shows the highest viscosity by several orders of magnitude in comparison to the AC and, even more so, in comparison to the GyC suspensions.

We combined the data on rheology and conductivity to address this issue and explore which particle type has the most favourable conductivity-flowability relation. To incorporate the results of our measurements at different pump rates, we plotted the conductivities *versus* the imposed stress. The stress was estimated by calculating the shear rate in the flow channel at the employed flow speed and extracting the corresponding viscosity from the rheological data. We estimated the shear rate in the rectangular channel using eqn (S3) and (S4) in the ESI.†^ ^^57^

The combined conductivity and rheology data are shown in Fig. 6b. For the AC and CB suspensions, conductivity indeed increases with stress, but not at the same rate. The onset for increasing conductivity in the AC graph is at a considerably lower stress than in the CB graph, showing that the relation between stress and conductivity is indeed dependent on particle type. Although AC does not give the most conductive slurry, it does show a higher increase in conductivity with lower increase in viscosity, and thus a more favourable trade-off between conductivity and flowability. In contrast to Fig. 6a, here AC appears to be the most suitable particle for a suspension electrode. A measurement with Ag NPs added to a 10 wt% suspension (with a ratio 3 : 1 AC : Ag) suggests that the Ag NPs can act as a conductive additive and increases the conductivity slightly without significantly influencing the flowability (see Fig. S4, ESI†).

Furthermore, Fig. 6b shows the conductivity at three different pump rates for each particle type and loading, with the data points at higher stress corresponding to those at higher pump rates. Although all suspensions are shear thinning in the region of shear rates (15–75 s^−1^) in which we conducted the conductivity measurements, faster pumping of AC suspensions increases the conductivity while decreasing the viscosity. This increased conductivity at faster pumping may be caused by more frequent collisions between particles or more collisions with the current collector at higher flow rates. The trend is different for the CB electrodes. These show an optimum in conductivity at the middle flow rate for most CB loadings, and the highest concentration CB (15 wt%) even causes the conductivity to drop for increased flow rate. We expect that this effect is caused by the interplay between more frequent collisions due to increased flow rate, the breaking of conductive networks when exceeding their yield stress,^45^ and a higher conductivity dependence on conductive networks due to lower surface area and capacitance compared to AC.

Finally, the GyC suspensions are a special case, showing a similar viscosity at 40 wt% as AC at 15 wt% and almost no conductivity in the tested loading range. Although much higher concentrations can be used at high flowability, the conductivity is inferior to AC even for similar stress.

### AC and CB give good modelled reaction distributions

**Fig. 7:**
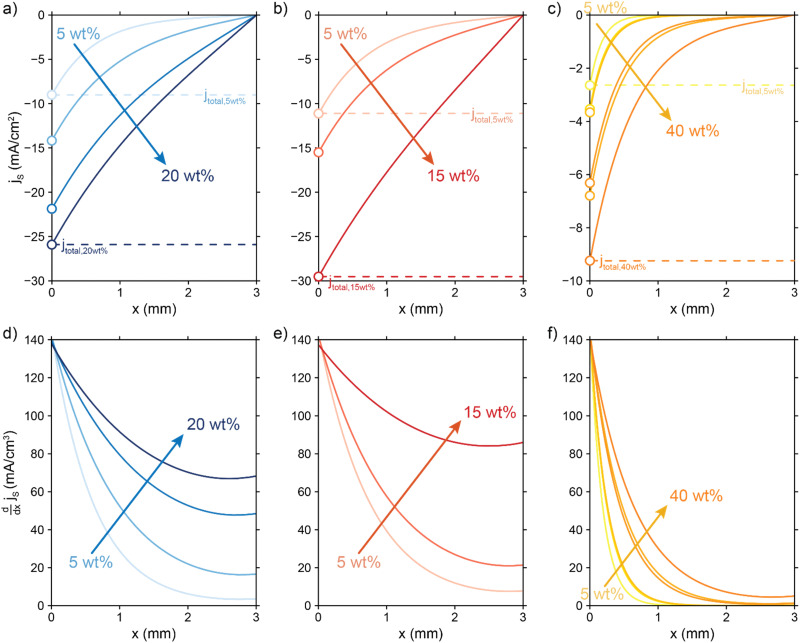
Modelled local current densities (top figures show *j*_S_, *j*_L_ can be found *via j*_L_ = *j*_S_|_*x* = 0_ − *j*_*S*_|_*x*_) and interfacial currents (bottom figures) throughout the electrolyzer channel for (a) and (d) AC, (b) and (e) CB and (c) and (f) GyC slurries. The highest currents and best interfacial current distribution can be achieved in 20 wt% AC and 15 wt% CB suspensions, of which the AC suspension is the most applicable due to higher flowability. The CO_2_ reduction reaction is localized near the current collector in all GyC suspensions. All simulations have been run at an 
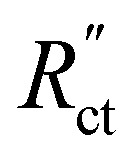
 of 10 Ω cm^3^ and an applied potential of −1.5 V *versus* the membrane (see ESI,† for remaining input values).

We implement the measured conductivities for all carbon types and loadings in the TLM, to determine the expected local current density and how well each suspension would be suited for use in a CO_2_ electrolyzer. We used the particle–particle resistances (*R*_p–p_) found with EIS and the ion conductivity of the electrolyte, adjusted with the Bruggeman equation (see ESI†), to define 
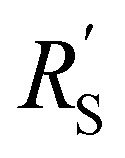
 and 
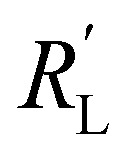
 in the TLM. We calculated 
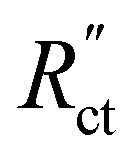
 with^50,58^8
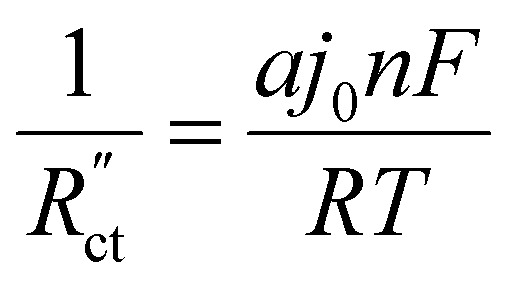
in which *a* is the ratio of surface area per volume, *j*_0_ the exchange current density (estimated as shown in ESI†), *n* is the number of electrons transferred in the reaction, and *F*, *R*, and *T* are the Faraday constant, universal gas constant and the temperature. For clarity, Fig. 7a–c show the local current density in the solid phase only. The intersection with the *y*-axis gives the total current density, which yields the liquid current density *via j*_L_ = *j*_total_ − *j*_S_.^59^

The total current shifts to larger values with higher carbon loadings due to lowered total resistance, showing that the loss of electrolyte volume and thus electrolyte conductivity is lower than the gain in solid conductivity upon raising the particle concentration. This is a direct consequence of a relatively low electric conductivity of suspensions (<8 mS cm^−1^, Fig. 6) compared to the ionic conductivity that can be reached at high electrolyte concentrations (44 mS cm^−1^ at 0.5 M KHCO_3_).

We saw that an even reaction distribution can be achieved if all three resistances are in the same order of magnitude, or 
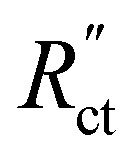
 is the limiting resistance. Fig. 7 shows the modelled local current densities (Fig. 7a–c) and the corresponding slopes (Fig. 7d–f) of AC, CB, and GyC suspensions at different carbon loadings. The values for 
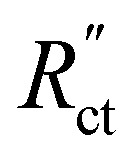
, 
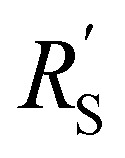
 and 
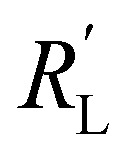
 used in these simulations are listed in Table S3 (ESI†). 
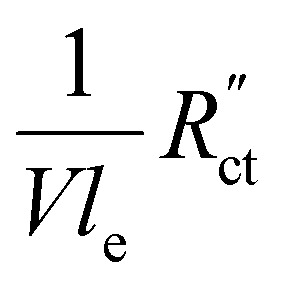
 and 
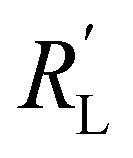
 are of the same order of magnitude in all situations, causing 
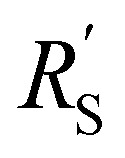
 to be the determining factor in how well the faradaic current is distributed over the channel. In case of AC and CB suspensions, the particle loading can be increased sufficiently to lower 
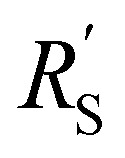
 into the same order of magnitude as 
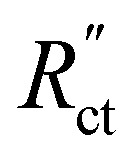
 and 
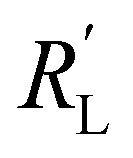
, resulting in a more linear decrease in current density through the channel (Fig. 7a and b) and a relatively constant slope (Fig. 7d and e). This indicates that even reaction distributions and a significant faradaic current throughout the whole channel can be achieved in suspensions of 15 and 20 wt% of AC, and 15 wt% of CB.

The resistances are even better matched at a lower electrolyte concentration (0.1 M, see Fig. S6 in ESI†). This presents a trade-off: when lowering the electrolyte concentration, the total current is lower, but the current is more evenly distributed over the thickness of the cell, which could allow a higher faradaic efficiency for CO_2_ conversion products. We can extrapolate the TLM results to higher current densities by applying a higher voltage (Fig. S7 and S8 for 0.5 and 0.1 M KHCO_3_, respectively, ESI†). This results in a similar shape for all carbon types and concentrations as for the original simulation at −1.5 V. This shows that the reaction distribution is mostly dependent on the ratio of 
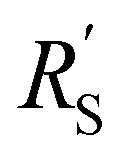
 and 
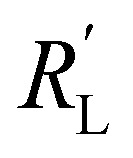
, and is not negatively influenced at higher voltages.

The less conductive GyC suspensions results in a much more localized current near the current collector and a lower total current density. The GyC conductivity is too low to drive the reaction deeper into the channel, even at very high loadings of 30 and 40 wt%. Therefore, we expect that CO_2_ reduction can benefit from a suspension electrode consisting of AC or CB particles, of which AC is the most applicable due to its higher flowability.

### Suspension electrodes show low selectivity for CO_2_ reduction

**Fig. 8:**
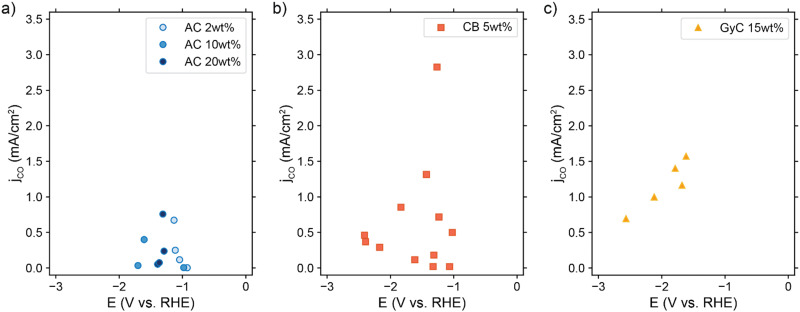
Resulting partial CO current densities in (a) AC, (b) CB, and (c) GyC suspensions in 0.5 M KHCO_3_, in which ¼ of the solid content consisted of Ag NPs. The cell setup contained 3 mm thick flow channels, separated by a Selemion anion exchange membrane. In each experiment, a graphite or glassy carbon plate current collector was used, and the liquid flow rate was varied between 9 and 18 mm s^−1^ (see Table S4 for additional experimental conditions, ESI†).

We experimentally assessed several suspension compositions in our CO_2_ electrolyzer setup. We show the achieved partial CO current densities in Fig. 8. Although all suspensions show some activity for CO_2_ reduction, they produce considerably larger amounts of H_2_ (see Table S4 and Fig. S10, ESI†). We reached the highest partial CO current density of 2.8 mA cm^−2^ in one experiment with 5 wt% CB, but in general the 15 wt% GyC suspension gave the most consistent trend in performance with the highest partial CO current density at 1.6 mA cm^−2^. This is surprising, as we expected the GyC suspensions to have the lowest performance due to the significantly lower conductivity. Additionally, we expected to see a clear trend in performance with increased AC loading, based on our TLM results. Instead, all suspensions give a similar partial CO current density, with no differences in low and high conductivity, as can be seen clearly from the AC graph (Fig. 8a) in which the 2 and 20 wt% AC suspensions reach roughly the same CO current density.

Due to this lack of trend in CO production with increased conductivity, we suspect that a different issue is outweighing the importance of suspension conductivity. This can be a number of engineering issues. For example, the average CO-selectivity is in the order of AC < CB < GyC, which follows the same trend as (1) the specific surface area of the powders, and (2) the concentrations of several metal contaminations in the powders (see Fig. S11, ESI†). This makes us suspect that the large amount of active sites provided by the carbon particles and metal contaminations catalyze the HER at the expense of CO_2_ reduction. Although high-surface area carbons are often used for adsorption of CO and other compounds, the high FE for H_2_ included in Table S4 (ESI†) shows that adsorption of CO (and other CO_2_ reduction products) is not significantly lowering the *j*_CO_ that we observe. Additionally, we suspect that our system suffers from a poor CO_2_ supply into the flow channel because of two likely causes. (1) Sparging CO_2_ into the reservoir may be too slow a saturation method to keep up with the CO_2_ consumption rate, causing the bulk CO_2_ concentration to drop over time.^60^ And (2) vortices indicating backflow were visible near the outlet inside the flow channel during experiments with the slightly more transparent suspensions. Therefore, we suspect that the viscous suspensions prevent efficient flushing with fresh (CO_2_-rich) electrolyte in our flow channel design. This hypothesis is supported by an increase in partial current density up to 2.7–3.3 mA cm^−2^ when using a 5 wt% AC suspension in combination with a smaller current collector area (Table S5, ESI†). Using a smaller electrode area at the same current density lowers the CO_2_ consumption and diminishes issues like slow CO_2_-resaturation in the reservoir and ineffective flushing of the flow channel with fresh electrolyte.

Although the TLM predicts two out of three suspension types to have sufficient conductivity for good performance, the practical issues described above are the likely cause for the inconsistency between the TLM predictions and the experimental CO_2_ electrolysis results and complicate the engineering of good suspension electrodes. As a result, the suspensions could not match the performance of state of the art GDEs, which can reach current densities of −200 mA cm^−2^. Comparing GDE-based and suspension-based CO_2_ electrolyzers, both technologies possess advantages and drawbacks in their operation. The silver loading per geometrical area of our system (7.5 mg cm^−2^) is slightly higher than in typical carbon-based GDEs (1 mg cm^−2^) before optimization. A lower Ag content (AC : Ag = 10 : 1) at 20 wt% solids produced similar low CO production (Table S6, ESI†) which suggest that the amount of Ag is not critical. Hence, the total amount of silver in suspension electrode may be optimized to similar quantities to those in GDEs and significantly lower than in alternative technologies like silver-based GDEs that consist almost completely (97%) of silver.^61^ Additionally, GDEs are complex structures to construct and they suffer from stability issues like carbon-degradation and electrowetting.^26–30^ Suspension electrodes can be produced from cheap carbon powders and the Ag catalyst can be incorporated by simple mixing, but these capacitive materials often contain contaminations that catalyze the HER to compete with CO_2_ reduction, and their flowability and stability during long-term operation are still under investigation.^62,63^ Flowable electrodes with a solid content of 5–20 wt%, depending on the particle type, are used throughout literature without significant clogging issues.^63,64^

## Conclusions

We modelled the local current densities in suspension electrodes with a transmission line model (TLM), and experimentally determined the electrode performances for CO_2_ reduction. Ideally, the faradaic reaction is distributed evenly over the whole depth of the flowable electrode. We used the model to study the required conditions to achieve this situation. We varied the ratio of solid and liquid phase resistances, in combination with high and low charge transfer resistance. The reaction is most evenly distributed when either charge transfer is the dominant resistance, or all three resistances are of a similar magnitude. When the charge transfer resistance is significantly lower than the solid and liquid phase resistivities, the reaction is always localized at the edges, losing the benefits of using a suspension electrode.

Choosing a highly conductive suspension is therefore crucial for the electrolyzer performance. Although the conductivity is most easily improved by increasing the carbon loading, this also significantly affects the viscosity. Unfortunately, the maximum carbon loading that maintains flowability limits the conductivity to 8 mS cm^−1^. Measuring the conductivity and rheology of small (CB) and larger irregularly shaped (AC) particles, and spherical (GyC) particles showed that the most viscous slurries do not necessarily yield the most conductive suspension. The relation between stress and conductivity is not linear and demonstrates that activated carbon has the highest conductivity when compared at equal stress, closely followed by carbon black.

When using experimentally obtained conductivities in the TLM, a good reaction distribution for the more conductive carbon materials is predicted. Instead, suspensions with carbon materials that feature lower conductivity should induce reactions only close to the current collector. Consequently, our modelling results predict the best catalytic performance in 20 wt% AC suspensions or 15 wt% CB.

However, our experiments showed no trends in achieved partial CO current density with carbon loading or conductivity, while we reached the best catalytic performance with j_CO_ of 2.8 and 1.6 mA cm^−2^ with the least conductive suspensions (5 wt% CB and 15 wt% GyC). These contradicting results may have been caused by several engineering limitations, such as flow cell design, metal contaminations in the carbon powders, or poor CO_2_-saturation of the electrolyte. We suspect that the CO_2_ reduction is too sensitive to contaminations, competing hydrogen evolution at the large surface area of the carbon, and CO_2_ dissolution limitations.

Although we achieved poor performance for CO_2_ electrolysis, our modelling results suggest that suspension electrodes can be applied in other mass transfer limited reactions. This could be a step towards intensifying electrochemical conversion processes that currently suffer from low limiting currents and are not sensitive to competing reaction and contaminations.

## Author contributions

All authors have given approval to the final version of the manuscript.

## Conflicts of interest

The authors declare no competing financial interests.

## Supplementary Material

YA-003-D3YA00611E-s001
