# Supplementary material for: Practical potential of suspension electrodes for enhanced limiting currents in electrochemical CO2 reduction
Source: Energy Adv. 2024 Mar 15;3(4):841–53. doi: 10.1039/d3ya00611e (PMC11025499; doi:10.1039/d3ya00611e)
Supplement: YA-003-D3YA00611E-s001 [file YA-003-D3YA00611E-s001.pdf]

Electronic Supplementary Material (ESI) for Energy Advances.
This journal is © The Royal Society of Chemistry 2024

Supporting Information

Practical Potential of Suspension Electrodes for Enhanced Limiting Currents in Electrochemical CO_2 Reduction

Nathalie E.G. Ligthart[1], Gerard Prats Vergel[1], Johan T. Padding[2], David A. Vermaas*[1]

[1] Department of Chemical Engineering, Delft University of Technology, Delft, The Netherlands

[2] Department of Process and Energy, Delft University of Technology, Delft, The Netherlands

Email: D.A.Vermaas@tudelft.nl

Contents

Modelling 1

Investigating influence of reaction and conduction resistances 1

Measuring suspension conductivity and flowability 2

Modelling reaction distribution in real suspensions 5

CO_2 reduction experiments 9

Modelling

The parameters that were kept constant and used in all TLM simulations are shown in Table S1.

Table S1. Parameters that were kept constant in all TLM situations.

Parameter	value	unit
$\boldsymbol{l_e}$	3	mm
$\boldsymbol{A_C}$	8.16	cm^2
$\boldsymbol{C'_{dl}}$	50	F/cm^3
$\boldsymbol{f}$ (with $\boldsymbol{\omega = 1\,rad \cdot f}$)	10^{-9}	s^{-1}
$\boldsymbol{V_{app}}$	1.5	V

Investigating influence of reaction and conduction resistances

The parameters that were varied to evaluate the influence of the ratio of volumetric charge transfer resistance $R_{ct}^{"}$ to the solid and liquid phase resistances R_S' and R_L' on electrode utilization are summarized in Table S2. The unit of $R_{ct}^{"}$ is Ω·cm^3, (as it represents the charge transfer resistance per surface area) while the unit of R_S' and R_L' is Ω/cm (as it depends on the thickness of the channel). To make the units match, we need to normalize $R_{ct}^{"}$. The values of $R_{ct}^{"}$ and $R_{S,L}'$ can be compared by adding a factor of

$$\frac{R'_{S,L}}{R_{ct}^{"}} = \frac{\frac{1}{j_{S,L}A_C dx}}{\frac{1}{ja}} = \frac{a}{A_c dx} = \frac{\frac{A_c}{A_c l_e}}{A_c l_e} = \frac{1}{A_c {l_e}^2} = \frac{1}{V l_e} \quad (S1)$$

Where $a = \frac{A_c}{A_c l_e}$, $dx = l_e$ is the thickness of the flow channel, and V is the volume of the flow channel. In our system the factor $\frac{1}{V l_e}$ is close to unity at 1.36 cm^{-4}.

Table S2. Suspension parameters used to find the influence of the ratio between reaction and conduction resistance on electrode utilization.

Ratios	R''_{ct} (Ohm·cm^3)	R'_S (Ohm/cm)	R'_L (Ohm/cm)
$\frac{1}{Vl_e}R''_{ct} \sim 100 R'_{S,L}$			
$R'_S = 99\%\ of\ R'_S + R'_L$	1000	19.8	0.2
$R'_S = 50\%\ of\ R'_S + R'_L$	1000	10	10
$R'_S = 1\%\ of\ R'_S + R'_L$	1000	0.2	19.8
$\frac{1}{Vl_e}R''_{ct} \approx R'_{S,L}$			
$R'_S = 99\%\ of\ R'_S + R'_L$	10	19.8	0.2
$R'_S = 50\%\ of\ R'_S + R'_L$	10	10	10
$R'_S = 1\%\ of\ R'_S + R'_L$	10	0.2	19.8
$\frac{1}{Vl_e}R''_{ct} \sim 0.01 R'_{S,L}$			
$R'_S = 99\%\ of\ R'_S + R'_L$	0.1	19.8	0.2
$R'_S = 50\%\ of\ R'_S + R'_L$	0.1	10	10
$R'_S = 1\%\ of\ R'_S + R'_L$	0.1	0.2	19.8

Measuring suspension conductivity and flowability

Each suspension was mixed at a shear rate of 2000 s^{-1} before each measurement and between different shear rates. Pre-mixing was performed for 2 minutes, each shear rate of interest was applied for 3 minutes, and the suspension were again mixed at 2000 s^{-1} for 1 minute between each shear rate, in case of AC and GyC suspensions. This was changed to 3, 4 and 2 minutes, respectively, in case of CB suspensions. The results of such a measurement is shown in Figure S1 for 10 wt% AC, CB, and GyC suspensions in 0.5 M $KHCO_3$. The highest stress plateaus correspond to the high-shear mixing modes at 2000 s^{-1}. The average values of the flat regions in the lower stress measurements, and the corresponding standard deviations, were used to produce Figure 5 in the manuscript.

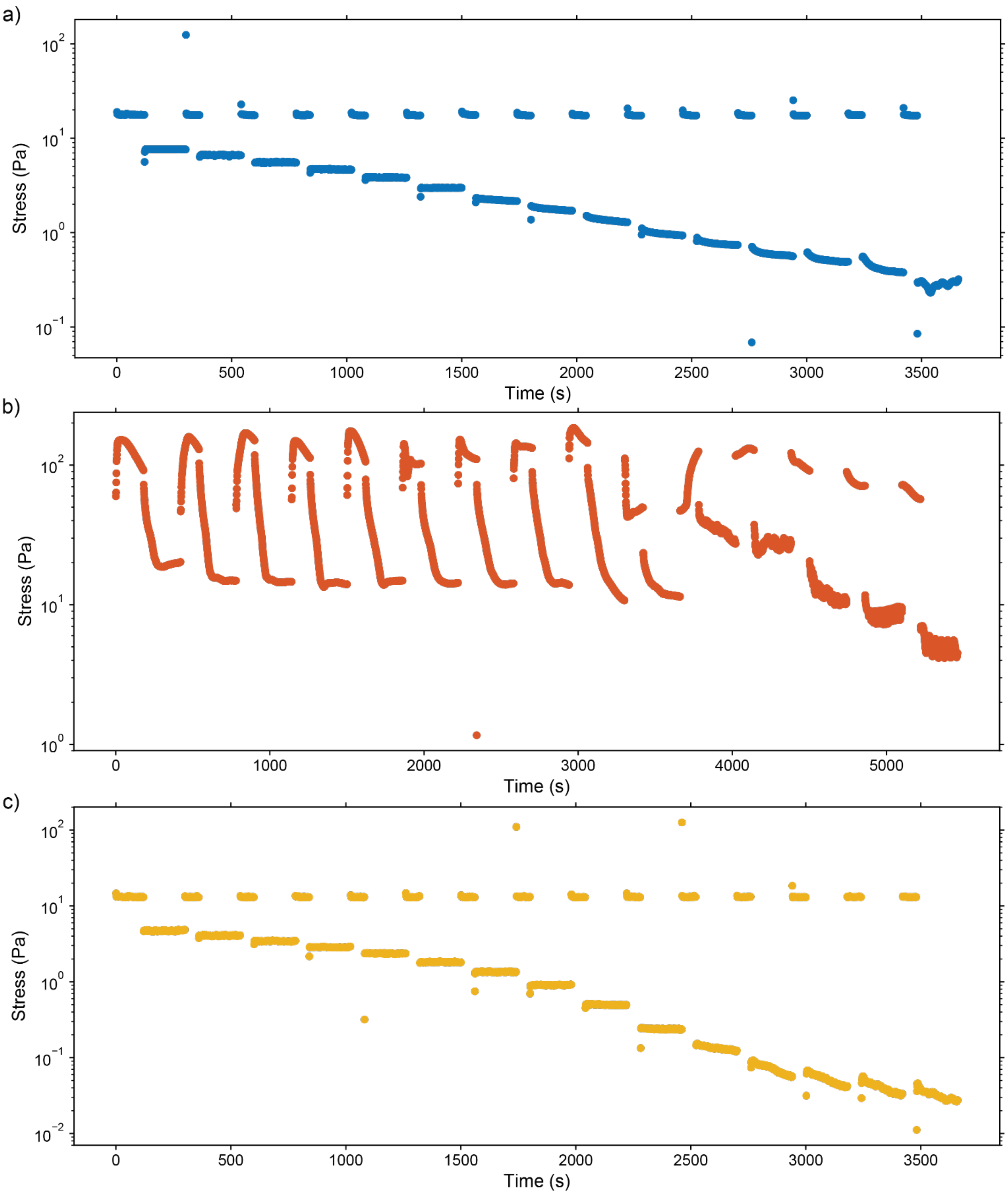

Figure S1. Measured stress over time at applied shear rates between 1000 and 2 s^{-1} for 10 wt% a) AC, b) CB, and c) GyC suspensions in 0.5 M $KHCO_3$. The suspensions were pre-sheared at 2000 s^{-1} before each new shear rate to prevent memory and sedimentation effects. The values of the applied shear rates can be found in Figure 5 in the main paper.

The solid phase conductivity was calculated from the particle resistance (R_{p-p}), that were obtained by fitting the EIS data to the equivalent circuit described in Figure 3 of the manuscript, and cell dimensions via

$$\sigma = \left(R_{p-p}\frac{A_C}{l_e}\right)^{-1} \quad (S2)$$

Some EIS results and fits for 10 wt% AC, CB, and GyC suspensions in 0.5 M $KHCO_3$ are shown in Figure S2.

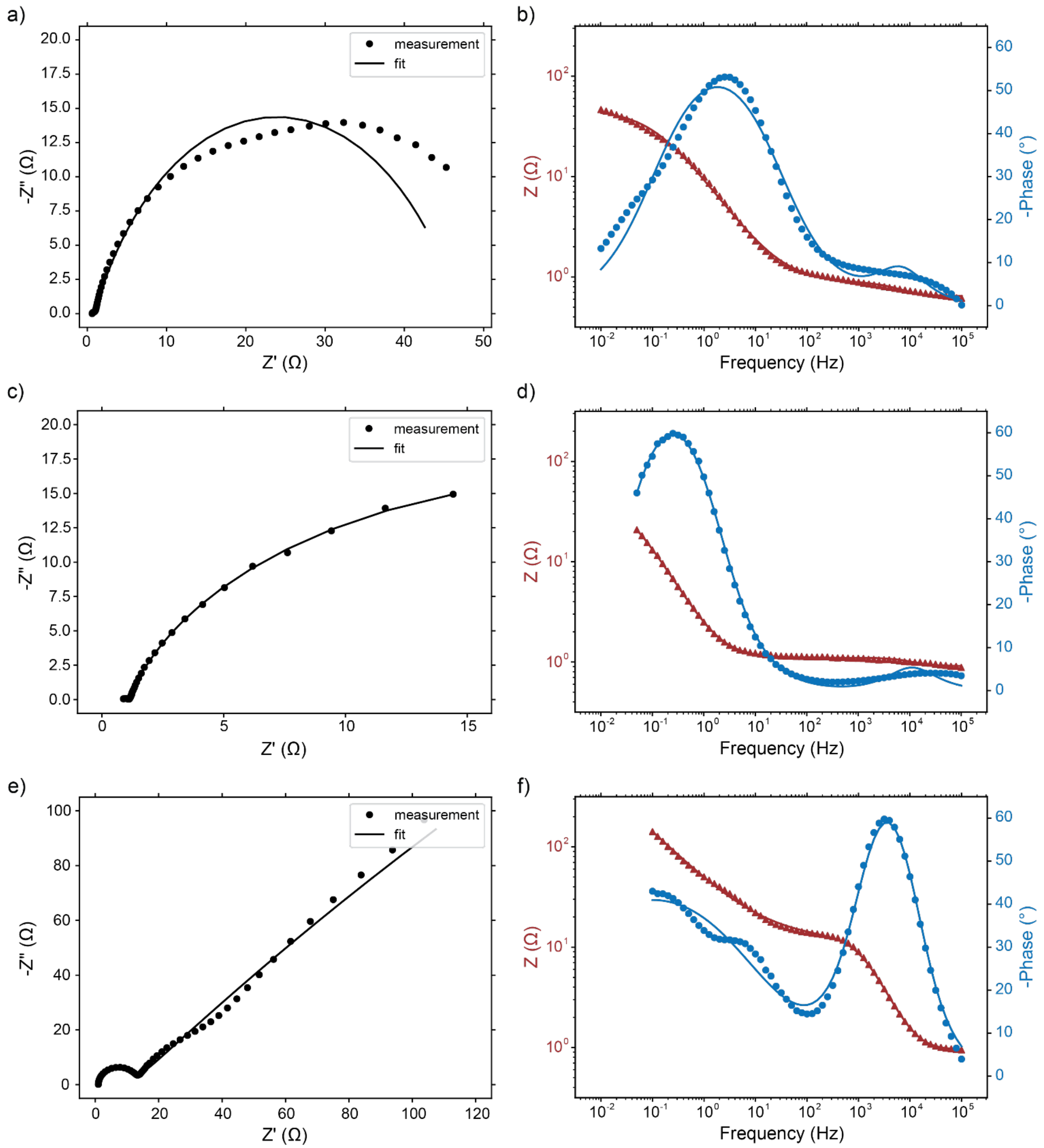

Figure S2. Typical EIS measurements and fitting, represented in Nyquist (left) and Bode (right). Results are shown for 10 wt% AC (a/b), CB (c/d), and GyC (e/f) suspensions in 0.5 M $KHCO_3$.

These results were combined with rheology data to produce Figure 6. The rheology measurements were not performed at the exact wall shear rates exerted by the suspensions inside the flow cell. We estimated the average wall shear rate $\dot{\gamma}$ in the rectangular channel with equations (S3) and (S4).[1]

$$\dot{\gamma} = \frac{QP\lambda}{8A^2} \qquad (S3)$$

$$\lambda = \frac{24}{\left(\left(1 - 0.351\frac{b}{a}\right)\left(1 + \frac{b}{a}\right)\right)^2} \qquad (S4)$$

which is valid when b/a < 1.0, where a and b are the lengths of the long and short sides, respectively. Q is the volumetric flow rate, P is the wetted perimeter, and A the cross-sectional area of the flow channel.

In order to plot the measured conductivity against the stress under flow conditions, we plotted all measured viscosities at shear rates between 2 and 100 s^{-1} and fitted a power function through these datapoints, as shown in Figure S3. We estimated the viscosity during pumping by inserting the relevant wall shear rate (resulting from equations (S2) and (S3)) into this function. Finally, the wall shear stress was calculated by multiplying the viscosity with the shear rate. These results were used in Figure 6b.

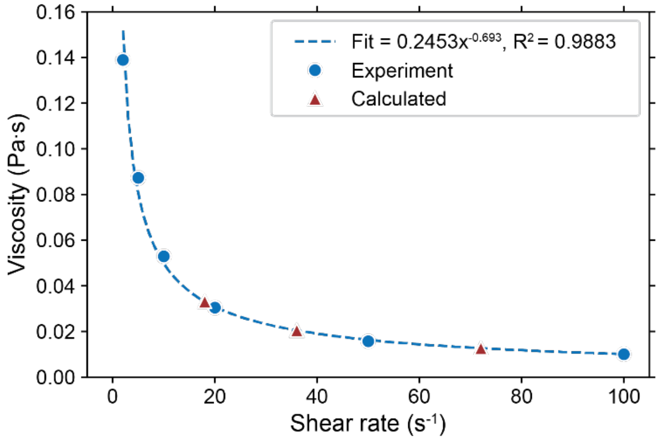

Figure S3. Measured viscosities of a 10 wt% AC suspension in 0.5 M $KHCO_3$ in the stress regime relevant for flow inside the CO_2 electrolysis cell. These datapoints and fit were used to calculate the stress at the flow rates used during the EIS experiments to produce Figure 6 in our paper.

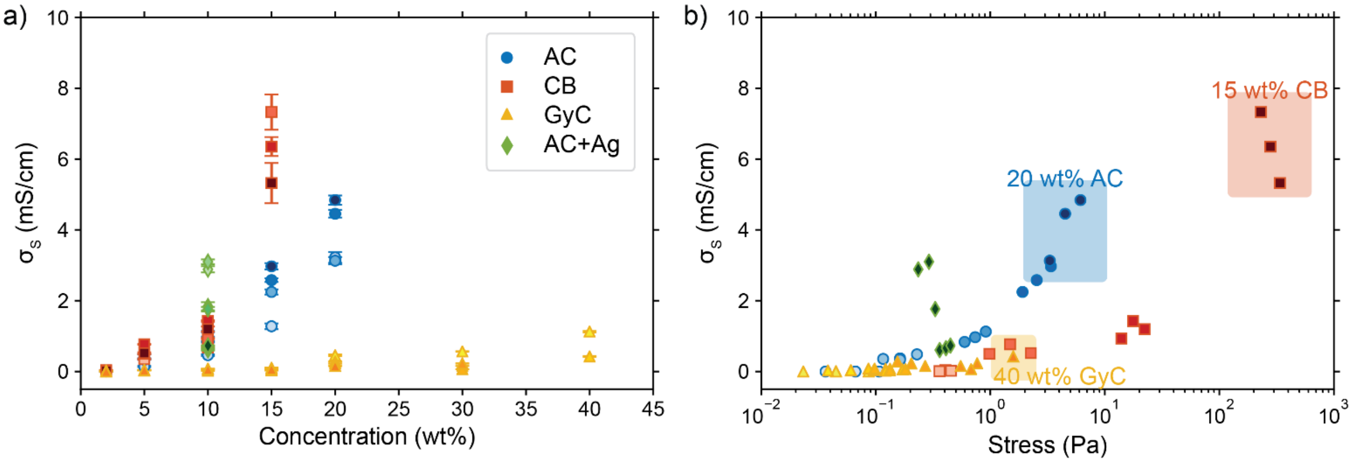

Figure S4. Influence of adding Ag NPs to a 10 wt% solids (3:1 AC:Ag) suspension in 0.5 M $KHCO_3$ on a) the suspension conductivity, and b) the relation between stress and conductivity. Note that adding Ag NPs increases conductivity while the stress remains approximately constant.

Modelling reaction distribution in real suspensions

The solid (R_S') and liquid (R_L') resistances per unit length used in the simulations for the real carbon suspensions can be calculated with[2]

$$R_S' = \frac{R}{l_e} = \frac{1}{A_C \sigma} \tag{S5}$$

in which R is the solid resistance, l_e the thickness of the channel, A_C is the cross-sectional area of the electrode and σ is the conductivity of the solid phase. We inserted R_{p-p} for R to account for the voids and changeability of the electrically conductive carbon networks. All calculated values for R_S' can be found in Table S3.

The values for R_L' were calculated in a similar manner with

$$R_L' = \frac{R}{l_e} = \frac{\tau}{p A_C \sigma_L} \tag{S6}$$

$$\tau = p^{-\frac{1}{2}} \tag{S7}$$

in which σ_L is the electrolyte conductivity, p is the porosity and τ is the tortuosity given by the Bruggeman relation, which are included to account for the solid fraction in the electrolyte, which is not ionically conductive.[3] We used conductivities of 0.044 and 0.010 S/cm for the 0.5 and 0.1 M $KHCO_3$ electrolytes, respectively[4] (calculated at 0.5 and 0.1 M $KHCO_3$ with 30 mM CO_2). We calculated the porosity with the volume fraction of carbon in water, for which we weighed a known volume of 10 wt% AC, and CB suspensions, assuming a water density of 0.997 g/cm3. This resulted in densities of 1.9 g/cm3 for AC and 1.8 g/cm3 for CB. We used a density of 1.5 g/cm3 for the GyC particles.[5] After which the porosity can be calculated as $p = 1 - \varphi_{carbon}$, with φ_{carbon} the volume fraction of carbon in the suspension. The resulting values for R'_L are shown in Table S3.

We calculated R''_{ct} with equation (9) in our paper and estimated the exchange current from the Tafel plots shown in Figure S4. We used the overpotential range in which we observed CO formation to make a linear fit and extract the exchange current, given by the intercept with the y-axis. This resulted in two R''_{ct} values of 4.6 and 13.3 Ohm·cm[3]. We used 10 Ohm·cm[3] in our suspension simulations.

Table S3. Suspension parameters used to find the influence of the ratio between reaction and conduction resistance on electrode utilization.

Carbon loading (wt%)	R''_{ct} (Ohm·cm^3)	R'_S (Ohm/cm)	R'_L (Ohm/cm)	
**AC**			0.1 M $KHCO_3$	0.5 M $KHCO_3$
5	10	317.1	12.6	2.9
10	10	125.4	13.2	3.0
15	10	47.1	13.8	3.2
20	10	29.6	14.5	3.3
**CB**				
5	10	205.7	12.7	2.9
10	10	103.5	13.3	3.0
15	10	19.4	13.9	3.2
**GyC**				
5	10	3754.7	12.8	2.9
10	10	2119.2	13.5	3.1
15	10	1959.8	14.3	3.3
20	10	557.7	15.3	3.5
30	10	824.4	17.7	4.0
40	10	288.9	21.0	4.8

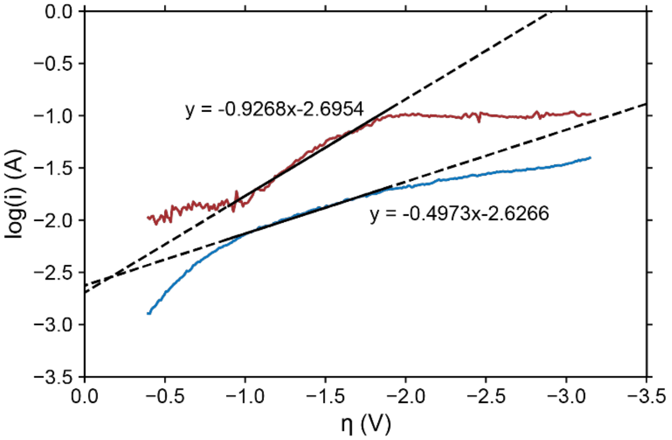

Figure S5. Tafel plots and linear fits for two CO_2 reduction experiments. The y-intercept gives the exchange current i_0 that was used for calculating R''_{ct}.

The simulation results for the suspensions in 0.1 M KHCO3 are shown in Figure S5. The interfacial current graphs are slightly flatter than the results in 0.5 M $KHCO_3$, indicating a better reaction distribution through the channel. However, the top figures show that the achievable total current is lower than in the higher concentration electrolyte, which is due to the higher total resistance. Although the interfacial current is spread out more evenly in all suspensions compared to the case with a higher electrolyte concentration, this does not significantly drive the reaction in GyC suspensions to the middle of the channel. The reaction in GyC suspensions is still highly localized near the current collector.

In addition, Figures S6 and S7 show that the simulated currents inside carbon suspensions in 0.5 and 0.1 M $KHCO_3$ can be increased by applying a higher potential. Running the TLM at -10 V increases the current density, while the shapes of the

graphs are very similar to the 0.5 and 0.1 M $KHCO_3$ cases at -1.5 V applied potential. This shows that the current distribution is mainly determined by the ratio of the solid and liquid phase resistances (R'_S and R'_L) instead of by the applied potential.

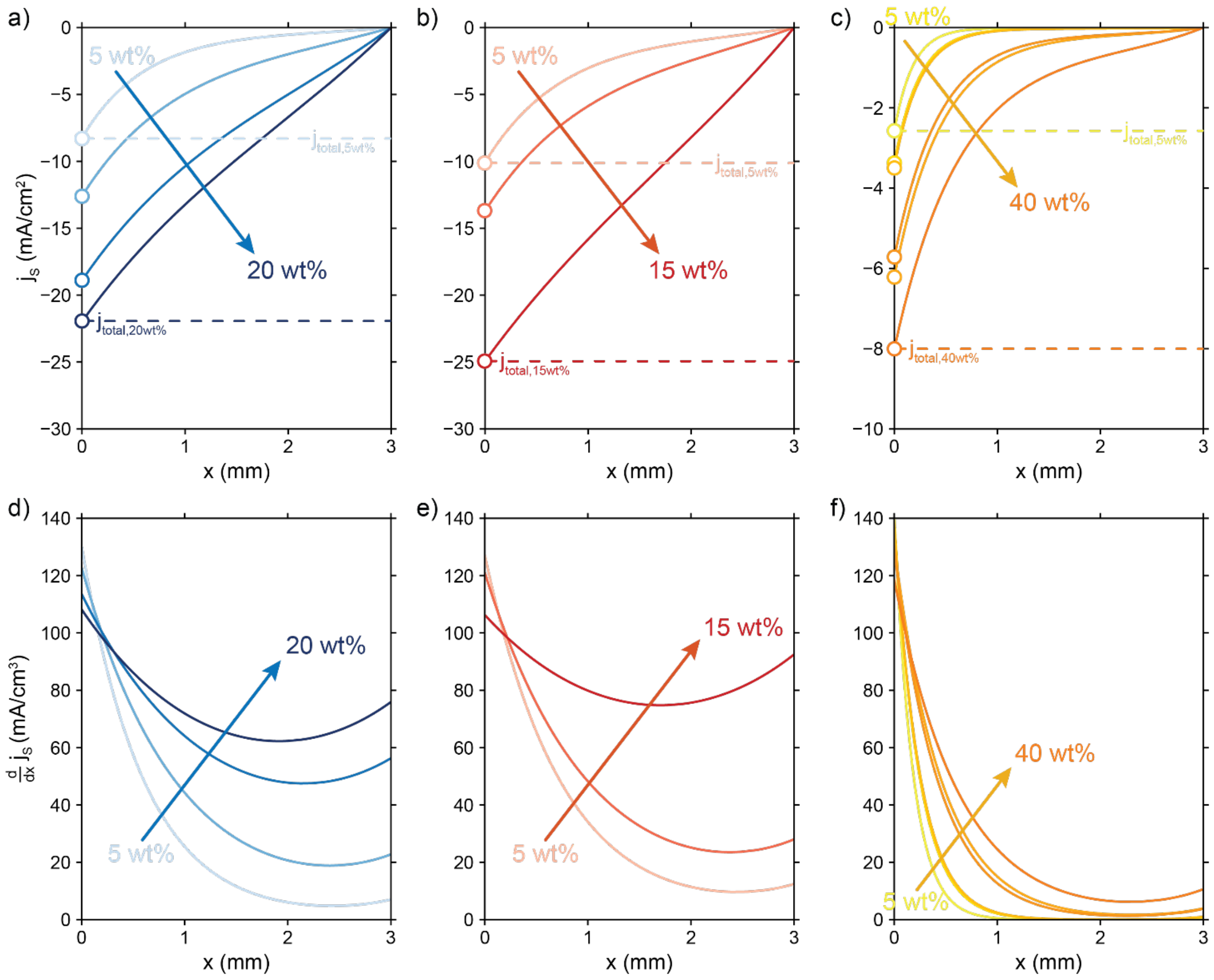

Figure S6. Modelled local current densities (top figures show j_S, j_L can be found via $j_L = j_S|_{x=0} - j_S|_x$) and interfacial currents (bottom figures) throughout the electrolyzer channel for a)/d) AC, b)/e) CB and c)/f) GyC slurries in 0.1 M $KHCO_3$. Although the total current are lower than in 0.5 M $KHCO_3$ electrolyte due to higher total resistance, the reaction distribution through the channel is slightly more uniform.

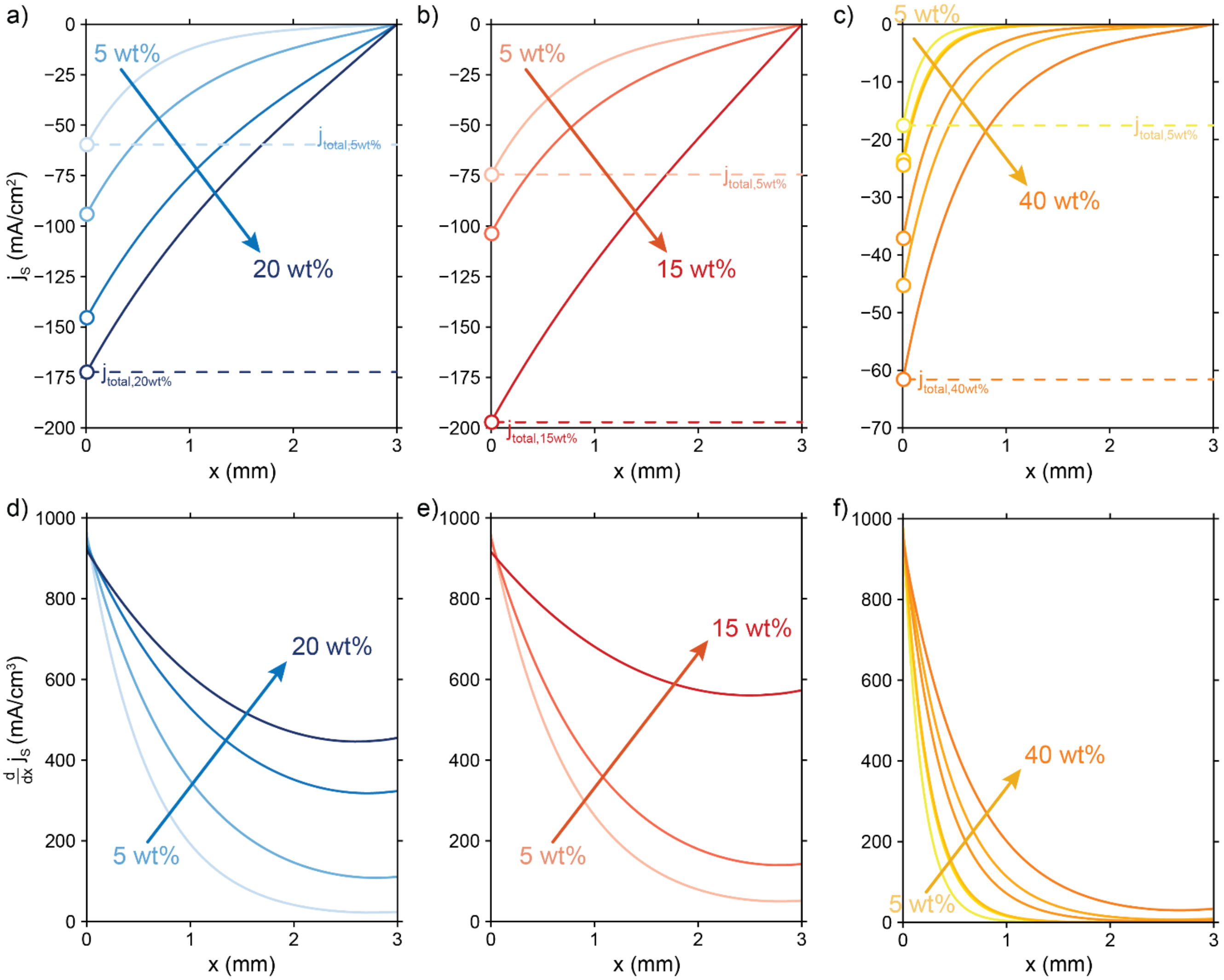

Figure S7. Modelled local current densities (top row shows j_S, $j_L = j_S|_{x=0} - j_S|_x$) and interfacial currents (bottom) in the electrolyzer channel for a)/d) AC, b)/e) CB and c)/f) GyC slurries in 0.5 M $KHCO_3$ at an applied current of -10 V to increase the current density.

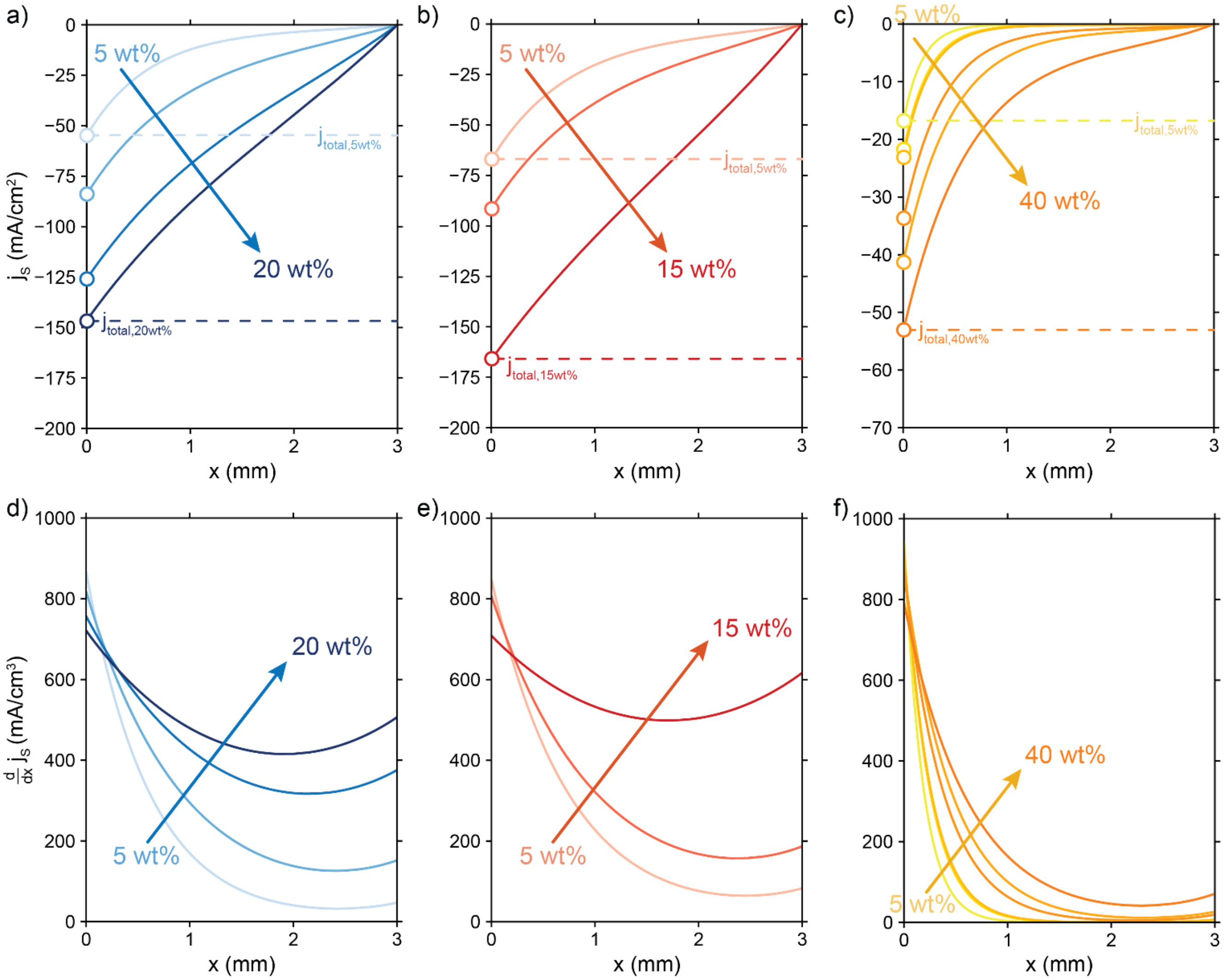

Figure S8. Modelled local current densities (top panels show j_S, $j_L = j_S|_{x=0} - j_S|_x$) and interfacial currents (bottom panels) in the electrolyzer channel for a)/d) AC, b)/e) CB and c)/f) GyC slurries in 0.1 M $KHCO_3$ at an applied current of -10 V to increase the current density.

CO_2 reduction experiments

All CO_2 reduction experiments were performed with the setup shown in Figure S9a. The system is airtight to allow for inline measurements of gas compositions. Figure S9b shows the cell configuration and dimensions. Table S4 shows all experimental conditions and results. The partial current densities towards H_2 are shown in Figure S10.

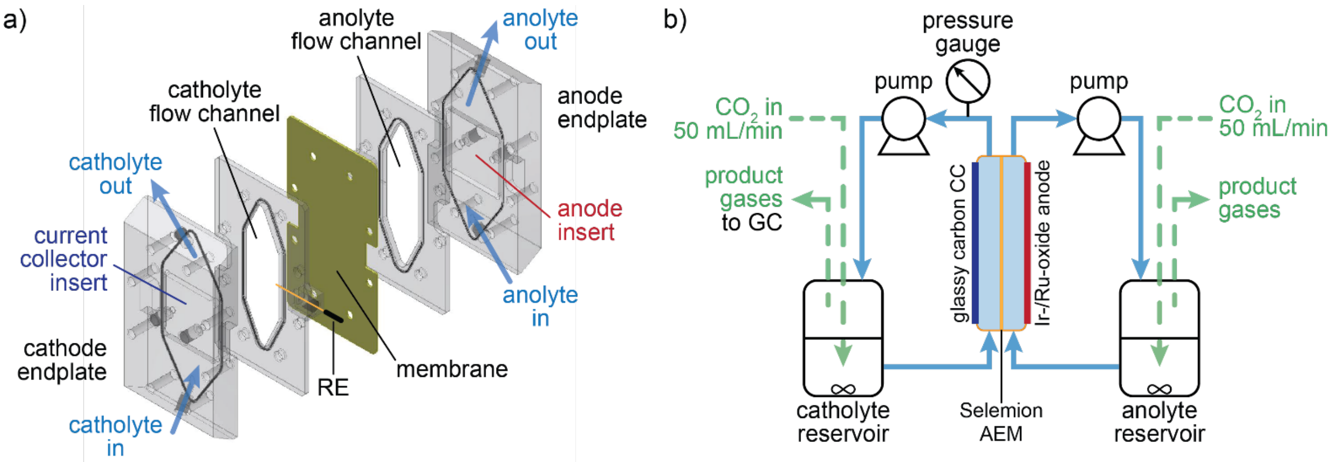

Figure S9. Illustration of the a) flow cell and b) setup used for CO_2 electrolysis.

Table S4. Experimental conditions and results for the performed CO_2 reduction experiments.

Carbon loading (wt%)	Run nr.	Current collector material	Flow rate (mm/s)	$j_{applied}$ (mA/cm^2)	FE CO (%)	FE H_2(%)	E vs. RHE (V)
**AC**							
2	1	Graphite	9	-25	2.7	80.8	-1.1
2	2	Graphite	9	-50	0.5	98.2	-1.1
2	3	Graphite	9	-50	0.2	99.6	-1.0
2	4	Graphite	9	-25	0.0	95.8	-0.9
10	1	Glassy carbon	18	-50	0.8	89.2	-1.6
10	2	Glassy carbon	18	-2.5	0.2	39.6	-1.0
10	3	Glassy carbon	18	-25	0.1	87.5	-1.7
20	1	Graphite	9	-25	3.0	84.1	-1.3
20	2	Graphite	9	-25	0.9	76.6	-1.3
20	3	Graphite	9	-50	0.1	89.8	-1.4
20	4	Graphite	9	-50	0.1	87.6	-1.4
**CB**							
5	1	Graphite	18	-50	0.4	95.4	-1.3
5	2	Graphite	9	-50	5.7	89.5	-1.3
5	3	Graphite	9	-25	2.0	92.2	-1.0
5	4	Graphite	9	-100	0.7	100.8	-1.2
5	1	Glassy carbon	18	-50	1.7	93.6	-1.8
5	2	Glassy carbon	18	-10	0.2	94.1	-1.1
5	3	Glassy carbon	18	-100	1.3	99.3	-1.4
5	4	Glassy carbon	18	-50	0.0	95.8	-1.3
5	1	Glassy carbon	18	-50	0.8	81.4	-2.4
5	2	Glassy carbon	18	-25	0.5	85.5	-1.6
5	3	Glassy carbon	18	-100	0.5	100.1	-2.4
5	4	Glassy carbon	18	-50	0.6	89.6	-2.2
**GyC**							
15	1	Glassy carbon	18	-50	2.1	87.2	-2.1
15	2	Glassy carbon	18	-100	0.7	85.2	-2.6
15	3	Glassy carbon	18	-25	5.8	66.1	-1.8
15	4	Glassy carbon	9	-25	4.9	68.7	-1.7
15	5	Glassy carbon	36	-25	6.5	65.3	-1.6

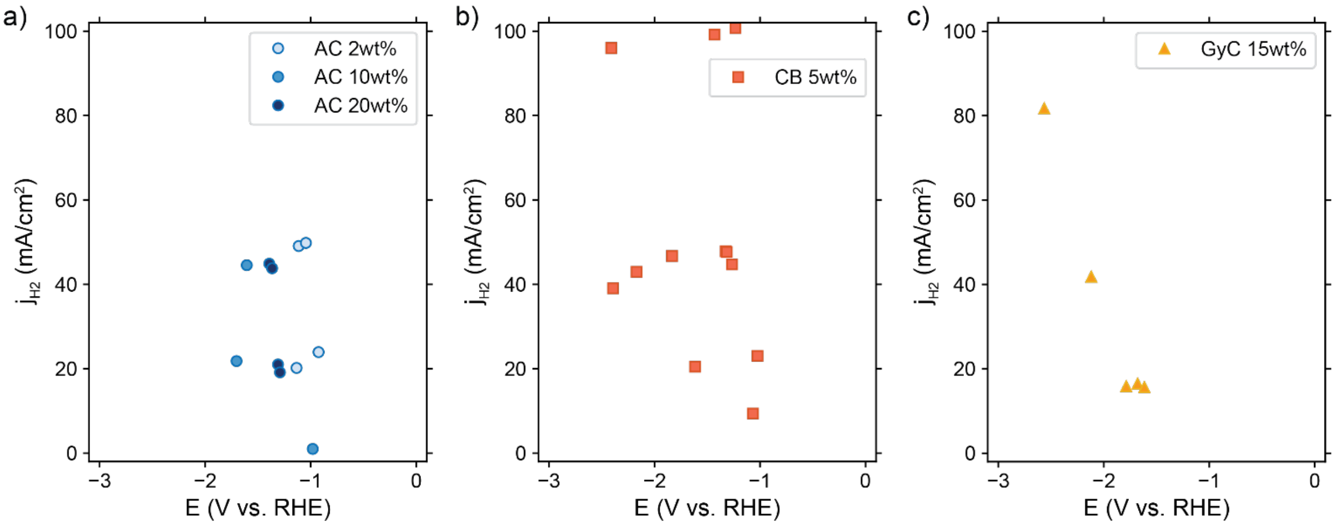

Figure S10. Resulting partial H_2 current densities in a) AC, b) CB, and c) GyC suspensions, under the reaction conditions listed in Table S4.

Table S5. Experimental conditions and results of CO_2R on an AC suspension using current collector with a 10 times smaller area.

Carbon loading (wt%)	Run nr.	Current collector material	Flow rate (mm/s)	$j_{applied}$ (mA/cm^2)	FE CO (%)	FE H_2(%)	E vs. RHE (V)
5	1	Graphite	9	-22	13.8	47.4	-1.2
5	2	Graphite	9	-44	6.1	74.7	-1.5

Table S6. Comparison of achieved partial CO current density for different amounts of Ag nanopowder per electrolyte weight.

Solids loading (wt%)	AC:Ag weight ratio	Ag concentration (g/g electrolyte)	$j_{applied}$ (mA/cm^2)	j_{CO} (mA/cm^2)
20	10:1	0.023	-25, -25, -50, -50	-0.8, -0.2, -0.1, -0.1
2	3:1	0.005	-25, -50, -50, -25	-0.7, -0.2, -0.1, -0.0

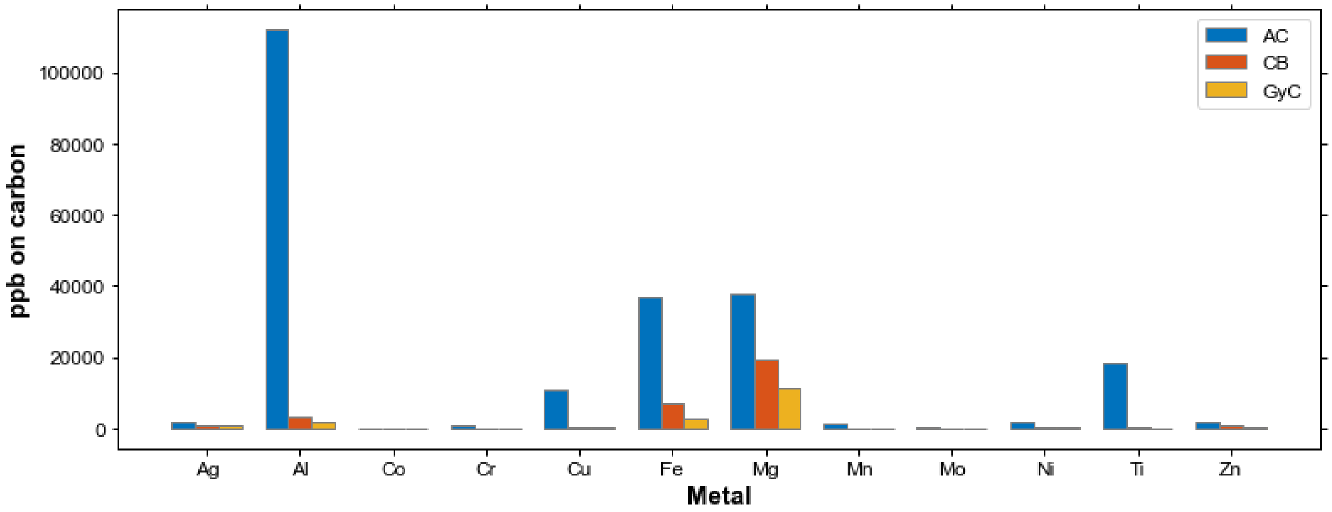

Figure S11. Metal contaminations on AC, CB, and GyC powders as measured with ICP-MS.

REFERENCES

1 Miller, C. Predicting Non-Newtonian Flow Behavior in Ducts of Unusual Cross Section. *Industrial & Engineering Chemistry Fundamentals* **11**, 524-528 (1972). https://doi.org/10.1021/i160044a015

2 Alfisi, D., Shocron, A. N., Gloukhovski, R., Vermaas, D. A. & Suss, M. E. Resistance Breakdown of a Membraneless Hydrogen–Bromine Redox Flow Battery. *ACS Sustainable Chemistry & Engineering* **10**, 12985-12992 (2022). https://doi.org/10.1021/acssuschemeng.2c02169

3 Gallagher, K. G. *et al.* Optimizing Areal Capacities through Understanding the Limitations of Lithium-Ion Electrodes. *Journal of The Electrochemical Society* **163**, A138-A149 (2016). https://doi.org/10.1149/2.0321602jes

4 *pH Calculator*, <https://www.aqion.onl/> (

5 Serp, P. in *Comprehensive Inorganic Chemistry II (Second Edition)* (eds Jan Reedijk & Kenneth Poeppelmeier) 323-369 (Elsevier, 2013).
